# Testicular yolk sac tumors in children: a review of 61 patients over 19 years

**DOI:** 10.1186/1477-7819-12-400

**Published:** 2014-12-29

**Authors:** Yi Wei, Shengde Wu, Tao Lin, Dawei He, Xuliang Li, Junhong Liu, Xing Liu, Yi Hua, Peng Lu, Guanghui Wei

**Affiliations:** Ministry of Education Key Laboratory of Child Development and Disorders; Key Laboratory of Pediatrics in Chongqing, CSTC2009CA5002; Chongqing International Science and Technology Cooperation Center for Child Development and Disorders, Room 806, Kejiao Building (NO.6 Building), No.136, 2nd Zhongshan Road, Chongqing City, Yuzhong District, China; Department of Pediatric Surgery, Children’s Hospital of Chongqing Medical University, Chongqing, China; Department of Respiratory Medicine, Children’s Hospital, Chongqing Medical University, Chongqing, China; Department of Pathology, Children’s Hospital of Chongqing Medical University, Chongqing, China

**Keywords:** Pediatric, Testicular yolk sac tumor, Diagnosis, Resection, Chemotherapy

## Abstract

**Background:**

To describe 19 years of clinical experience managing pediatric patients with testicular yolk sac tumors at the Chongqing Medical University Affiliated Children’s Hospital.

**Methods:**

This study involved a retrospective review of the records of 61 pediatric patients who presented with testicular yolk sac tumor at our institution between 1995 and 2014.

**Results:**

All patients presented with a painless scrotal mass. Serum alpha-fetoprotein (AFP) levels were elevated (n = 15). Ultrasonography identified the yolk sac tumors as solid masses. Color Doppler flow imaging showed rich blood flow inside and around the masses in 84.8% cases. X-ray of the scrotum showed no intrascrotal calcification (n = 38). Inguinal orchiectomy was performed in 60 patients, one case was treated with testis-sparing surgery. In 11 cases, radical dissection of the inguinal lymph nodes was performed. Histological analysis showed pathologies typical of yolk sac tumor including microcapsule and reticular structures, gland tube-gland bubble structures, an embryo sinus structure, and papillary structures. All patients received postoperative chemotherapy. Serum AFP levels returned to normal 1 to 2 months after surgery. No patients treated with surgery in our hospital relapsed.

**Conclusion:**

Testicular yolk sac tumor presents as a painless scrotal mass, increased serum AFP levels, and a solid mass on ultrasound. Chest radiography and abdominal ultrasound should be used to accurately stage the tumor. We advocate for inguinal orchiectomy for Stage I disease and postoperative chemotherapy to prevent recurrence in the ipsilateral or contralateral testis.

## Background

Testicular tumors are uncommon in children, comprising approximately 1% to 2% of all pediatric malignancies [1–4]. However, the incidence of testicular tumors in children is increasing, and associated morbidity has doubled during the last 40 years [4, 5]. Testicular yolk sac tumors account for 70% to 80% of prepubertal malignant testicular tumors and are the most common childhood testicular cancer [6, 7]. The prognosis of testicular yolk sac tumors is dependent on early detection and treatment.

Pediatric patients with testicular yolk sac tumors usually present with an asymptomatic scrotal mass early (Stage I) in the disease process. Evaluation of the solid scrotal mass includes: scrotal ultrasound; chest, abdominal, and pelvic computed tomography (CT); and determination of serum tumor marker levels such as alpha-fetoprotein (AFP) and beta-human chorionic gonadotropin (β-hCG). An elevated serum AFP level is closely associated with yolk sac tumors in more than 90% of patients [7].

The treatment of testicular yolk sac tumors is dependent on tumor stage and patient age. Resection and chemotherapy with or without retroperitoneal lymph node dissection (RPLND) is often used for children with elevated or rising AFP levels and/or retroperitoneal lymphadenopathy. In cases of recurrence or metastasis, the vast majority of pediatric patients can be successfully treated with chemotherapy [8, 9].

This report describes 19 years of clinical experience managing pediatric patients with testicular yolk sac tumors at the Chongqing Medical University Affiliated Children’s Hospital. These data will improve current knowledge of diagnostic and treatment processes for the disease.

## Methods

### Ethics statement

This study was approved by the Ethical Committee of Chongqing Medical University.

### Study subjects

This study involved a retrospective review of 61 patient records at the Chongqing Medical University Affiliated Children’s Hospital from 1995 to 2014. Inclusion criteria were: (1) presence of a painless scrotal mass; (2) suspected testicular tumor; and (3) underwent treatment for testicular yolk sac tumor. Exclusion criteria were: (1) mixed testicular tumors (yolk sac tumor with teratoma); or (2) no accurate pathological evidence of tumor.

Information extracted from patient records included: (1) age; (2) clinical manifestation (tenderness, obvious mass when ‘bearing-down’); (3) physical examination (cremasteric reflex, transillumination test); (4) personal and family medical history; (5) routine laboratory parameters and serum AFP levels; (6) preoperative imaging data, including ultrasonography of the testis and abdomen, color Doppler flow imaging (CDFI) of the testis, and plain scrotal X-ray; (5) treatment; (6) histopathologic diagnosis and analysis; and (7) follow-up, including abdominal CT imaging to detect evidence of metastasis.

### Management strategy

All tumors were surgically resected. Inguinal orchiectomy was performed in 60 patients. The lower abdomen was explored through a lateral incision, and the spermatic cord was clamped. The scrotum and the tunica vaginalis on the affected side were incised, and the testis was isolated. A frozen biopsy was immediately sent for histopathology. Complete excision of the mass and inguinal orchiectomy was performed based on the biopsy result and parental preference. If lymphadenectasis was found in the inguinal region, the inguinal lymph nodes were excised.

Tumors were staged based on the TNM Classification of Malignant Tumors published by the International Union Against Cancer in 2009 [2]: Stage I, primary tumors with no evidence of metastasis on clinical examination or imaging; Stage II, tumors with subphrenic lymph node metastasis; Stage III, tumors with mediastinal and supraclavicular lymph node metastasis.

All patients received postoperative chemotherapy.

### Histological analysis

Postoperative pathological examination was performed at the Institute of Pathology, Chongqing Medical University Affiliated Children’s Hospital. Samples were collected during surgery and fixed in 10% neutral formalin, dehydrated through graded alcohols, and embedded in paraffin. Sections of 4 μm were cut for histology, stained with hematoxylin and eosin (H&E), and examined using light microscopy.

Immunohistochemical staining included AFP, cytokeratin (CK), vimentin (Vim), placental alkaline phosphatase (PALP), epithelial membrane antigen (EMA), CD117, and Ki-67. A tissue was considered positive for a marker if >10% cells were stained. A tissue was considered negative for a marker if ≤10% cells were stained.

### Follow-up

Patients continued chemotherapy and were followed up in the inpatient department of our institution at postoperative 1 and 2 months. Subsequently, patients returned for follow-up visits once every 6 months for 48 months. Follow-up visits included routine blood tests, evaluation of biochemical indicators (serum AFP levels, liver and kidney function, cardiac markers, sex hormone levels), electrocardiography, and imaging (sonography of the scrotum and abdomen, X-ray).

### Statistical analysis

Statistical analysis was performed with GraphPad Prism 4.0 (San Diego, CA, USA). Data are expressed as mean ± SEM. Between group differences were analyzed with Student t-test. Significance was defined as *P* <0.05.

## Results and discussion

### Demographic and clinical characteristics of the patients

All patients presented with a painless scrotal mass; 26 occurred on the left side, 35 occurred on the right side. The masses were solid with a smooth surface and were obvious on ‘bearing down’. Six cases were misdiagnosed as hydrocele, four cases as inguinal hernia, two cases as testicular inflammation, and one case as adenoma. These cases were misdiagnosed for presenting with a painless scrotal mass, however, all were cleared with transillumination test, ultrasonography, and the frozen biopsy: (1) transillumination test result was positive for the six hydrocele and negative for the yolk sac tumors; (2) the four inguinal hernia were shown as intestinal canal-shaped structures on ultrasonography; (3) the two testicular inflammations were described as diffusely enlarged with rich blood flow, no space-occupying lesion were found inside on ultrasound; (4) ultrasound showed tumor tissue with adenoid structure beside normal testis and clear septum between tumor and testis, while no specific structure change in this case of adenoid carcinoma of testis. Diagnosis was confirmed by intraoperative frozen biopsy.

**Figure 1:**
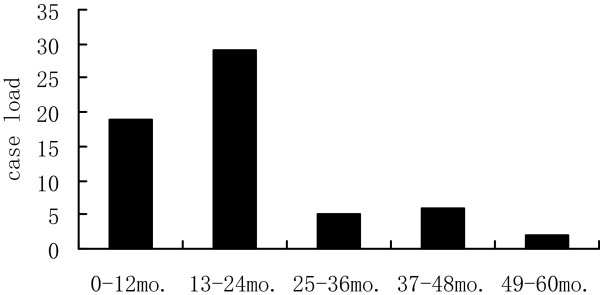
**The age distribution of the 61 pediatric patients suffering from testicular yolk sac tumors.**

Mean age at diagnosis was 1.5 years ± 1.0 years (range, 2 months to 4.5 years) (Figure  1). Mean time from presentation to diagnosis was 3 months 26 days ± 2 months 18 days (range, 2 days to 1 year).

Fifty-nine patients were diagnosed with Stage I testicular yolk sac tumor and no retroperitoneal metastasis, two patients were diagnosed with Stage II testicular yolk sac tumor and a retroperitoneal mass identified on ultrasonography and CT.

### Serum alpha-fetoprotein

**Figure 2:**
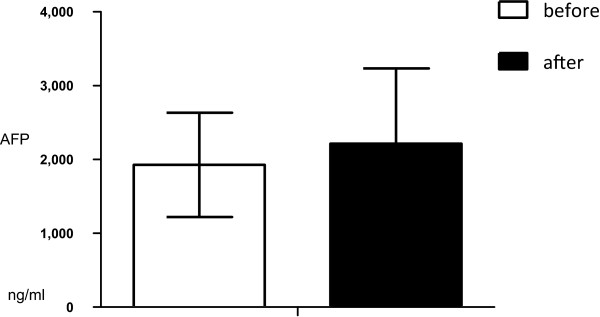
**Preoperative and postoperative serum AFP levels (n = 15; P = 0.679).**

Mean serum AFP level before surgery was 1,319.31 ng/mL ± 45.35 ng/mL (range, 49.9 to 14,900 ng/mL). Mean serum AFP level 14 days after surgery was 2,581 (04 ng/mL ± 216.61 ng/mL; range, 22.6 to 14,500 ng/mL). There was no significant difference between preoperative and postoperative serum AFP levels (n = 15; *P* = 0.679) (Figure  2).

### Imaging

Ultrasonography identified the yolk sac tumors as solid masses; focal masses were evident when the tumor replaced the entire testicle. The masses were described as hyperechoic in 8% of cases (5/61); hyperechoic and isoechoic in 46% of cases (28/61), isoechoic in 28% of cases (17/61); isoechoic and hypoechoic in 6.5% of cases (4/61), hypoechoic in 5% of cases (3/61), and ‘mixed abnormal echo’ in 6.5% of cases (4/61). In 14 cases, there was an anechoic area, which was identified as a hydrocele. Mean dimensions of the masses on ultrasound were 53 cm^3^ ± 19.6 cm^3^ (range, 1.5*1.1*0.7 to 9.4*8.8*5.1 cm).

**Figure 3:**
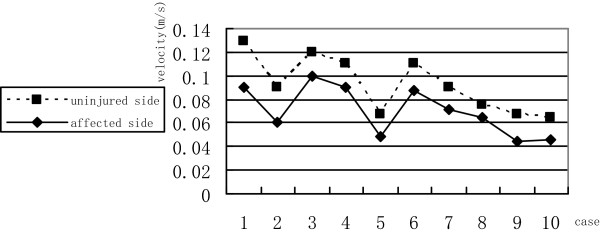
**Blood-flow velocity inside and around the testicular yolk sac tumors and the normal testes.**

CDFI showed rich blood flow inside and around the masses in 85.5% cases (52/61). Mean blood-flow velocity inside and around the masses was 0.071 m/s ± 0.018 m/s (range, 0.09 to 0.1 m/s). Mean blood flow inside and around the normal testes was 0.024 m/s ± 0.007 m/s (range, 0.018 to 0.039 m/s) which shows blood-flow velocity inside and around the masses is faster than it inside and around the normal testes (Figure  3).

X-ray of the scrotum was performed in 38 cases; no intrascrotal calcification was seen. In one case, chest radiography was used to examine a suspected lesion. In three cases, abdominal ultrasonography was used to investigate a huge retroperitoneal mass (n = 2) or necrosis (n = 1). In one case, CT revealed a lesion occupying the left retroperitoneal space that displaced the left kidney.

### Histological analysis

**Figure 4:**
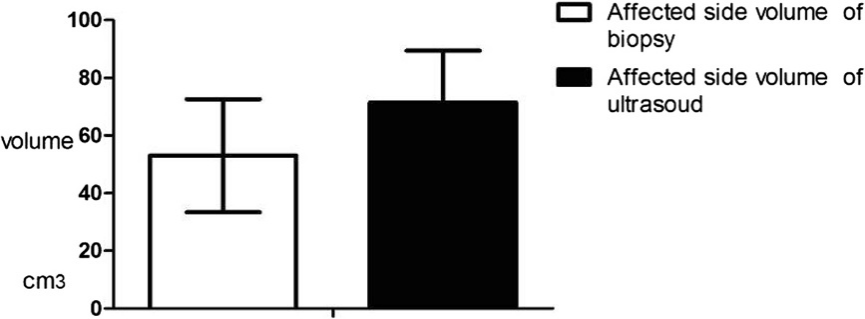
**Mean volumes of the testicular yolk sac tumors on ultrasound and following resection (P = 0.887).**

Pathological examination of the yolk sac tumors was performed in all cases. All tumors were solid masses; in some cases there were areas of necrosis and hemorrhage. Testicular enlargement was evident when the tumor replaced the entire testicle. Mean dimensions of the masses following resection were 71.44 cm^3^ ± 17.95 cm^3^ (range, 2*1.8*1.2 to 10.5*6.5*5.2 cm. The difference between the mean volumes of the solid masses on ultrasound and following resection was not significant (ultrasound: 53 ± 19.6 cm^3^ vs. biopsy: 71.44 ± 17.95 cm^3^; *P* = 0.887; Figure  4). Mean length of the spermatic cords was 5.45 cm ± 1.95 cm (range, 2.5 to 10 cm) and mean diameter of the spermatic cords was 1.53 cm ± 0.95 cm (range, 0.4 to 3 cm).

**Figure 5:**
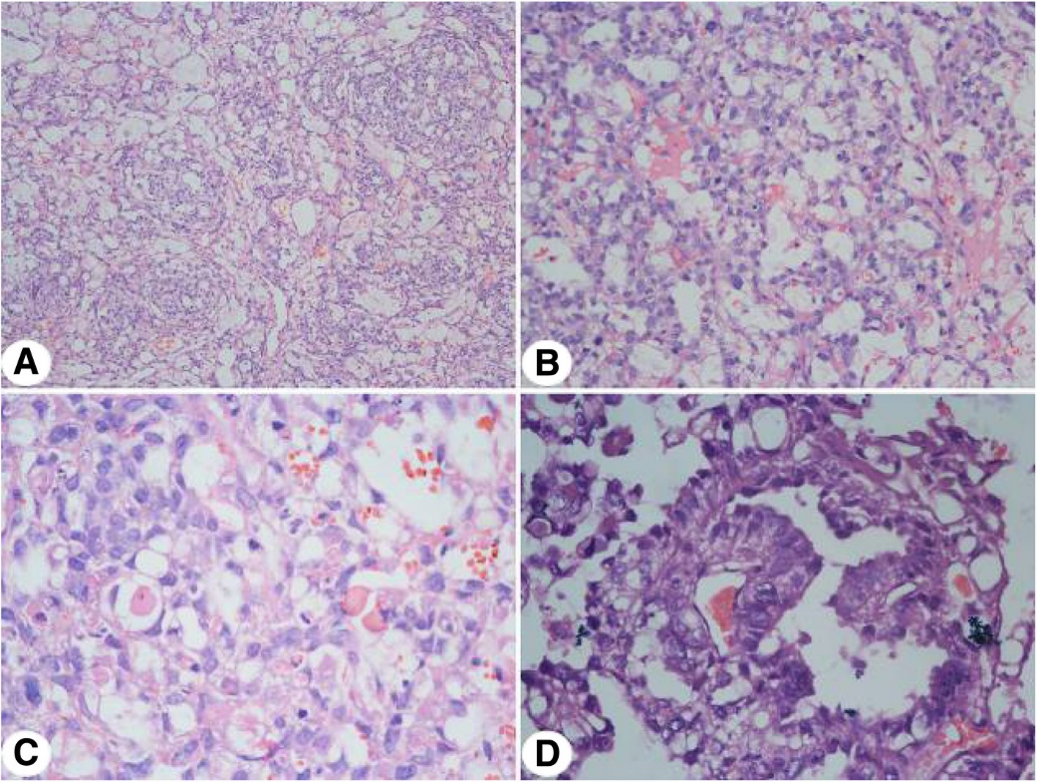
**Representative pathology of the testicular yolk sac tumors. (A)** Micro capsule and reticular structure (X200); **(B)** Gland tube-gland bubble structure (X200); **(C)** Acidophilic droplets (X400); **(D)** Schiller-Duval droplets (X400).

**Figure 6:**
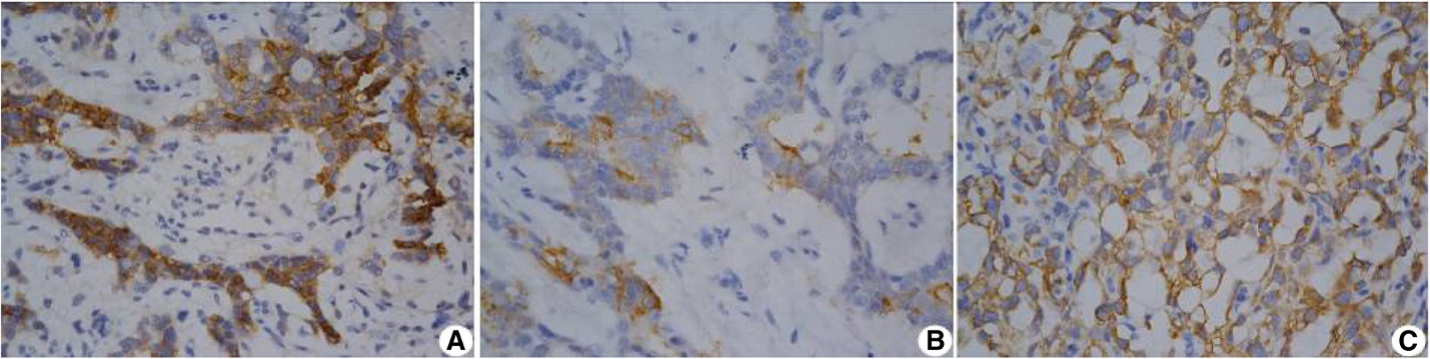
**Representative immunohistochemical pathology of the testicular yolk sac tumors. (A)** Alpha-fetoprotein (X400); **(B)** placental alkaline phosphatase (X400); **(C)** cytokeratin (X400).

Tumors were gray and grayish yellow. Histological micrographs demonstrated pathologies typical of yolk sac tumors: 32 cases showed a microcapsule and reticular structure, 14 cases showed a gland tube-gland bubble structure, one case showed an embryo sinus structure, and two cases showed a papillary structure (Figure  5). Acidophilic droplets were revealed in 32 cases and eosinophilic staining was seen in 12 cases. Immunohistochemical staining showed the positive expression rate for AFP was 100% (21/21), CK was 100% (21 /21), Vim was 66.67% (12/18), PLAP was 94.74% (18/19), EMA was 21.5% (4/19), CD117 was 100% (6/6), and Ki67 ranged from 20% to 90%. Two cases showed positive c-kit staining and one case was negative. Pathology representative of the 61 cases is shown in Figure  6.

### Management strategy

#### Surgery

Inguinal orchiectomy was performed in 60 patients, one case was treated with testis-sparing surgery. Of the 60 patients, 53 patients underwent complete resection of the primary tumor and inguinal orchiectomy at our institution. Seven patients underwent complete resection of the primary tumor and inguinal orchiectomy at another institution. Of these seven patients, two patients gave up treatment because of huge retroperitoneal mass under abdominal ultrasonography.

In 11 cases, radical dissection of the inguinal lymph nodes was performed because lymphadenovarix was found on preoperative ultrasonography and during physical examination. Of these, four patients were treated with inguinal mass-sparing surgery as there was no evidence of lymph node metastases.

#### Postoperative chemotherapy

Prior to 2009, there were no clear guidelines for administration of chemotherapy to pediatric patients with testicular yolk sac tumor. At this time, 14 patients received a combination of vincristine and dactinomycin, one patient received a combination of bleomycin, etoposide, and carboplatin, two patients received vincristine alone, and one patient received a combination of vincristine and carboplatin.

After 2009, 29 patients received a combination of cisplatinum, etoposide, and bleomycin (PEB), (Platinol: Days 1 to 5, Etoposide: Days to 3, Bleomycin: Day 4), and these patients repeat the course for 3 weeks and last for 3 to 5 weeks on average. One patient received a combination of carboplatin, etoposide, and bleomycin (JEB), and one patient received PEB and cyclophosphamide.

One to two months after the initiation of chemotherapy, 16% (5/31) of the patients at follow-up experienced anemia (minimum hemoglobin, 79 g/L) and leukopenia (minimum white blood cell count, 1.8×10^9^/L). Levels normalized following blood transfusion and myocardial preservation, allowing the patients to complete chemotherapy.

### Follow-up

Follow-up information was obtained for 50.8% (31/61) of patients. The average length of follow-up was 48 months. Of these, three patients experienced relapse. Overall survival rate was 100%, and there was no evidence of metastasis. AFP, chest radiography, and abdominal ultrasonography were used as follow-up indicators. In 16 patients, serum AFP level was normal after 2 years of follow-up. In all patients, chest radiography and electrocardiogram 1 and 2 months post surgery were normal. In three patients, ultrasonography revealed an inguinal mass. Two patients underwent inguinal mass resection; histology confirmed the absence of tumor cells. One patient underwent inguinal mass resection and hernia sac high ligation. The mass was no longer present at the next follow-up.

Table  1. Clinical characteristics of the 61 patients with testicular yolk sac tumor.

**Table 1:** **Clinical characteristics of the 61 patients with testicular yolk sac tumor**

	Case no.	Age (months)	Tumor	Pre-AFP (ng/mL)	Surgery	CTX	Follow-up (months)	AFP (ng/mL) (follow-up)	Surgery (follow-up)	CTX (follow-up)	Biopsy (follow-up)	Outcome (48 month average)
	1	6	YST	1,020	HIO	VCR + KSM	1	32.4	-	CBP + VP-16		NED
	2	17	YST	6,160	HIO	PEB	4	2.3-10.2	-	PEB		NED
	3	25	YST	>363	HIO	PEB	7	1.8-16.9	-	PEB		NED
	4	15	YST	>363	HIO	PEB	3	1.5-38.3	-	PEB		NED
	5	45	YST	151	HIO	PEB	3	1.7-3.7	-	PEB		NED
	6	6	YST	990	HIO	PEB	6	2.53-8.5	-	PEB + PVB		NED
	7	20	YST	>363	HIO	PEB	2	2.6-8.4	-	PEB + PVB		NED
	8	24	YST	102	HIO	PEB	5	2.3-3.2	-	PEB + PVB		NED
	9	18	YST	91.6	HIO	PEB	2	2.9-4.31	-	PEB + PVB		NED
	10	23	YST	14,900	HIO	PEB	2	1.8-17.5	-	PEB		NED
	11	23	YST	>363	HIO	PEB	2	8.65-40.2	-	PEB + PVB		NED
	12	15	YST	>363	HIO	PEB	2	1.88-5.05	-	PEB + PVB		NED
	13	7	YST	>363	HIO	PEB	2	9.6-24.8	-	PEB		NED
	14	13	YST	>363	HIO	PEB	2	Unknown	-	PEB		NED
	15	21	YST	Unknown	HIO	Unknown	2	1.78-5.49	-	PEB + PVB		NED
	16	21	YST	>363	HIO	PEB	3	4.2-15.5	-	PEB + PVB		NED
	17	12	YST	>363	HIO	PEB	2	19.7-63.5	-	PEB + PVB		NED
	18	54	YST	>363	HIO	PEB	2	3.3-169	-	PEB		NED
	19	39	YST	>363	HIO	PEB	2	2.7	-	PEB		NED
	20	50	YST	20.9	HIO	PEB	2	2.44-3.71	-	PEB		NED
	21	12	YST	>363	HIO	PEB	2	23.1-4.4	-	PEB		NED
	22	10	YST	>363	HIO	PEB	2	5.84-19.1	-	PEB		NED
	23	41	YST	>363	HIO	PEB	2	2.12-7.2	-	PEB		NED
	24	13	YST	>363	HIO	PEB	2	3.78-32.4	-	PEB		NED
	25	1	YST	>363	HIO	PEB	1	6.85	-	PEB		NED
	26	25	YST	142	HIO	PEB	2	2.76-5.35	-	PEB		NED
	27	10	YST	>363	HIO	PEB	1	2.52	-	PEB		NED
	28	15	YST		HIO	Unknown	3	3.34-23.1	-	CBP + VCR + BLM		NED
Relapse	29	21	YST	>363	HIO	PEB	2	3.75-11.9	Plan A excision + hernia sac high ligation	PEB	Match	NED
	30	14	YST	9,092.5	HIO	VCR + KSM	9th month	>400	Plan B	CBP + VCR + KSM	Match	NED
							10th month	Normal		VCR + KSM		NED
	31	23	YST	284	HIO	VCR	3rd month	909	Plan B	VCR + KSM + DDP	Match	NED
Stage II	32	23	YST	Unknown	TSS	VCR			-	Abandon		Unkown
	33	30	YST	Unknown	HIO	Unknown (outer court)	5th month	>4,000	-	Abandon		Unkown
	34-61	Lost to follow-up after the surgery										

Cases 15 and 33 treated outer court. Cases 1, 25, and 27 were followed up for 1 month. The other 28 cases were followed up as described in Table  2.

AFP: Alpha-fetoprotein; CBP: Carboplatin; DDP: Cisplatin; HIO: High inguinal orchiectomy; KSM: D-actinomycin; NED: No evidence of disease; Normal AFP: 0 to 12 ng/mL; Plan A: Scrotal mass excision + hernia sac high ligation; Plan B: Scrotal mass excision; VCR: vincristine.

Table  2. Clinical follow-up data of 28 patients treated with chemotherapy and no relapse.

**Table 2:** **Clinical follow-up data of 28 patients treated with chemotherapy and no relapse**

Case no.	Preoperative AFP	Postoperative AFP (1 month)	Postoperative AFP (2 months)
1-7	High	Normal	Normal
8-21	High	High	Normal
22	9,092	11	11
23	990	8.5	8.1
24	>363	6.85	Unknown
25	>363	2.52	23.1
26	>363	40.2	8,65
27	>363	24.8	9.6
28	>363	63.5	19.7

Dissection of inguinal mass and lymph nodes was carried out in case 22 due to a solid mass identified by ultrasonography. PEB was used in 17 cases (60%), PEB + PVB was used in nine cases (32%), CBP + VCR + BLM was used in one case (4%), and CBP + VP was used in one case (4%).

AFP: Alpha-fetoprotein; Normal AFP: 0 to 12 ng/mL.

## Discussion

Yolk sac tumors are the most common primary tumors of the testes in prepubertal patients [8]. The painless hard enlargement of a previously normal testis is the most frequent presentation of testicular yolk sac tumor. However, published reports of painless scrotal masses in pediatric patients are scarce [9], as most attention is given to painful scrotal swellings. Due to the non-specific clinical manifestations, signs, and symptoms of testicular yolk sac tumor, misdiagnosis is common. Parents of children with risk factors for testicular cancer, such as a history of cryptorchidism, microlithiasis, and a family history of cancer should be particularly vigilant [10]. Differential diagnosis should include inguinal hernia, hydrocele, and testicular inflammation using a transillumination test, ‘bearing down’, and clinical evaluation of tenderness. Previous studies show that the mean time from presentation to diagnosis in pediatric patients is 6 months for germ cell tumors and 18 to 24 months for non-germ cell tumors [11, 12]. This is substantially longer than the 3 months and 26 days reported here, suggesting that parents are becoming increasingly aware that the presence of a painless scrotal swelling in a child requires prompt medical attention.

Testicular yolk sac tumors frequently secrete high concentrations of AFP; therefore, AFP is considered important in the diagnosis and follow-up of the tumors [13]. Evidence suggests that infants aged younger than 1 year with serum AFP levels >100 ng/mL should be presumed to have a yolk sac tumor, and that serum AFP levels should be monitored postoperatively to indicate yolk sac tumor recurrence during follow-up. If AFP levels do not decrease by postoperative day 5, residual tumor or metastatic disease should be considered [13]. In the current study, serum AFP levels were measured 2 weeks after surgery in 15 pediatric patients treated for testicular yolk sac tumor. Although serum AFP levels remained elevated in five cases, scrotal sonography, abdominal sonography, and chest X-ray did not reveal metastasis. As there was no significant difference between preoperative and postoperative serum AFP levels in our patients, the reliability of elevated postoperative serum AFP levels for predicting recurrence and metastasis requires further investigation. No statistical difference was found in serum AFP level 2 weeks after yolk sac tumor resection compared with that preoperatively. Two aspects may be referred to this result: (1) for patients just after resection, the decline of AFP level needs time, and that may not be exactly its half-life time (4 to 6 days); (2) naturally, mean age of patients with yolk sac tumor is relatively low, serum AFP is relatively high. No decrease or even elevation of serum AFP level is not the sound evidence for failure in surgery or possibility of metastasis. Thus, a long-term follow-up visit is highlighted.

Ultrasound is the most common method for diagnosis of testicular tumors; it can also be used to differentiate benign and malignant testicular lesions [14, 15]. Importantly, ultrasound can distinguish intra- and paratesticular lesions. The majority of intratesticular lesions are malignant, while more paratesticular lesions are benign. In the current study, ultrasonography and CDFI identified the tumors as solid masses with a rich blood supply. CT should be used for yolk sac tumor staging and identification of metastasis.

We used intraoperative analyses of frozen-sections for routine evaluation of testicular yolk sac tumors to provide evidence for orchiectomy. Paraffin-embedded sections were used for histologic analysis after surgery. Histopathologic analyses of solid yolk sac tumors frequently show microcystic/reticular patterns. In a previous study reporting the histology of 52 solid yolk sac tumors, 75% were microcystic/reticular, 35% were glandular, and 25% were myxoid [16]. These data are in accordance with the findings reported here. Solid foci consisted of sheets of cells with abundant cytoplasm and intercellular basement membrane deposits.

In the past, management of prepubertal testicular tumors was based on experience with adult testicular cancer. At present, clinicians agree that prepubertal testicular neoplasms differ greatly from postpubertal lesions. Traditionally, high inguinal orchiectomy was recommended for testicular yolk sac tumor because of the cancer’s aggressive nature [17]. More recently, testis-sparing surgery has been suggested as a safe and efficacious choice for patients with a testicular tumor of its diameter less than 2 cm [18]. In the current study, only one case had a tumor that was less than 2 cm. The remaining 60 patients were treated with high inguinal orchiectomy.

RPLND may prevent metastasis in patients with testicular yolk sac tumors [2, 19, 20]. However, there is still controversy concerning the use of RPLND in patients classified as Stage 1. We do not advocate for RPLND for the following reasons: (1) metastasis in patients with testicular yolk sac tumors is rare, and it is currently unclear whether lymphangiectasis results in metastasis; (2) the associated extensive surgical trauma may cause lymphatic fistula, enteroplegia, pulmonary atelectasis, and ejaculation incompetence in later life; and (3) the therapeutic effect of chemotherapy with or without lymph node dissection is not significantly different. Importantly, none of the patients in this study experienced relapse, which suggests RPLND may not be required for the routine management of Stage I testicular yolk sac tumors.

Chemotherapy is recommended for the treatment of yolk sac tumor. Platinum-based cancer drugs have recognized efficacy [21]. However, some evidence suggests that chemotherapy has no influence on the therapeutic effect of orchiectomy on Stage 1 testicular yolk sac tumors, and that systematic chemotherapy may be omitted for Stage 1 patients aged less than 1 year with no recurrence or metastasis and serum AFP stabilizing at normal levels [19]. We routinely administered chemotherapy (PEB); and the project were carried out as (Platinol: Days to 5, Etoposide: Days to 3, Bleomycin: Day 4); serum AFP levels returned to normal and no recurrence or metastasis was found beside the cases treated with surgery out our court without regular chemotherapy. In one case, chemotherapy was omitted at first. At 8 months of follow-up, a mass was found in the inguinal region on the affected side and serum AFP level was elevated. These observations suggest postoperative chemotherapy should be applied to pediatric patients with testicular yolk sac tumor. Routine blood tests, hepatorenal function analyses, and evaluations of myocardial marker and sex hormone levels have a role in identifying the side effects of postoperative chemotherapy. In older patients, cryopreservation of semen may be considered if there is a possibility of infertility.

This study improves current knowledge of diagnostic and treatment processes for testicular yolk sac tumor. However, it is associated with a number of limitations. First, some patients’ records were incomplete. Second, parent/patient preferences influenced management decisions, which may have affected outcomes. Future studies with longer follow-up are required to fully inform the safe and effective management of testicular yolk sac tumor.

## Conclusions

In summary, testicular yolk sac tumor presents as a painless scrotal mass and an increased serum AFP level (>100 ng/mL). Based on our clinical experience, we recommend ultrasound as a reliable method for preoperative localization and evaluation of testicular yolk sac tumor. Chest radiography, CT, and abdominal ultrasound should be used to accurately stage the tumor. We propose high inguinal orchiectomy for Stage I disease, RPLND for Stages II or III, and more weeks are needed for the older patients (>18 months) with higher AFP levels to fall to normal, which could be classified as Stage II. But intraoperative histopathological evaluation of frozen sections should guide the extent of surgery. Postoperative chemotherapy (PEB) should be used to prevent recurrence in the ipsilateral or contralateral testis. AFP and ultrasonography is considered important in the follow-up of the tumors, and the initial AFP monitoring is proposed in 2 to 3 weeks after operation. Long-term follow-up (>48 month) is recommended.
